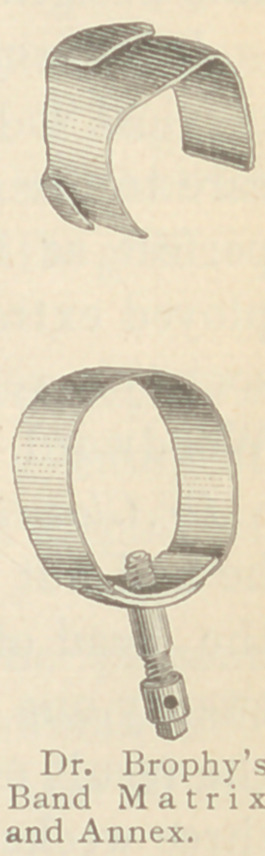# New York Odontological Society

**Published:** 1886-03

**Authors:** 


### NEW YORK ODONTOLOGICAL SOCIETY.

Reported Expressly for the Independent Practitioner.

The parlors of the New York Academy of Medicine were crowded Tuesday evening, February the 9th, it being the regular monthly meeting of the New York Odontological Society. The usual number was largely added to by members of the profession from Boston, Philadelphia, Chicago and other cities, making the meeting one of unusual interest.

Dr. J. Morgan Howe called the meeting to order and appointed a committee to escort the newly-elected president, Dr. Bogue, to the chair.

Dr. Bogue read a short address, stating the influence which the society exerted, not only throughout our own country, but abroad, and expressed his hopes and belief that it would be still more active.

Dr. Griffin, a physician from a New York hospital, presented a patient, a man about twenty-five years of age, with "a tooth in his nose." A right central incisor had taken an erratic course in process of development, the coronal portion finding its way into the right nostril, which had become much dilated by the disturbing influence of the annoying intruder, while the apex of the root appeared at a point where the crown should have been, just projecting through the gum. The doctor stated that Dr. Alexander Mott, Sr., had performed an operation in the patient's mouth when a child, and he imagined that the tooth germ was disturbed at that time, hence its eruption in the position found.

Dr. Raymond showed a storage battery and electric headlight for making examinations, or treating the mouth in the evening, or on dark days. This consisted of a little electric disk attached to a wire frame which was placed on the head like spectacles, the disk of light being on the forehead, thus leaving both hands free.

Dr. Brophy, of Chicago, presented a mouth mirror with lens attached, which could be moved at almost any angle, thus focusing the light on the point being operated upon.

Dr. Northrop passed around some surgeon's lint, which he stated had been given him by Dr. Delos Palmer, who used it for its excellent absorbent properties.

Dr. J. Morgan Howe presented some matrices from England.

Dr. S. G. Perry explained his new hand-piece for the Bonwill engine. To reduce the friction he employed a fine fish-line in place of the cable in general use, and to prevent slipping, the edge of the large (or fly) wheel was covered with a strip of thin rubber dam, as suggested by Dr. Wardwell.

Dr. W. W. Allport, of Chicago, called the attention of the society to some matrices designed by Dr. Mattison, to be used in putting in amalgam fillings.

Dr Niles, of Boston, stated he had a case of congenital cleft palate, in a very young patient, which, he thought, might be reduced by continual lateral pressure, and asked Dr. Kingsley if such treatment had ever been resorted to.

Dr. Kingsley replied that it had, by Dr. Sayre, fifteen minutes after the child came into the world. Though a very difficult operation, Dr. Kingsley thought that if sewed up at a very early age nature would so assist as in time to give a very good result. As to the lateral pressure, he thought this, if applied, would very much contract the arch.

The regular paper of the meeting was read by Prof. T. W. Brophy, of Chicago, upon the use of the matrix in filling teeth. He advocated their employment because it obviated the necessity for removing so much of the cervical portion of the tooth in the preparation of the cavity. Retaining pits, which so often endanger the vitality of the tooth, are not required, and the base of the cavity can be left smooth and even, thus avoiding the danger from friable walls left for the purpose of retaining the filling. The anchorage can be made upon the surface where there is no danger of leakage.

Concerning the materials with which to fill teeth, he stated that the difference between cohesive and non-cohesive gold consisted in a film of some substance which prevents the cohesion of each piece to the preceding one. When he desires to use soft gold, he places it in a drawer and subjects the pellets to the fumes arising from a few drops of ammonia. This renders it soft and velvety, and prevents its too ready cohesion. If such gold be annealed the ammonia is driven off, and it becomes again cohesive. Of tin, he believes that its efficiency is not due to any antiseptic qualities which it may possess, but to its softness and the readiness with which it may be brought into adaptation with the walls of the cavity.

He recommended the use of a combination of tin and gold, and quoted from the article by Prof. Miller in the INDEPENDENT PRACTITIONER for August, 1884, concerning its admirable properties. It is readily inserted, works with extreme softness, and it seems a most excellent material for the preservation of teeth. After insertion a crystallization takes place, which makes the filling nearly or quite as hard as an amalgam one, and gives an excellent wearing surface.

He believes that the great advances made in operative dentistry in late years are due more to the rubber dam and the matrix than to any other appliances at our disposal. The credit for the introduction of the matrix is due to Dr. Louis Jack, of Philadelphia. Many operators have failed in obtaining the best results from it through a want of familiarity in its manipulation. He himself had abandoned its use once because he had failed with it to obtain perfect margins, but his failure was due to his incomplete knowledge of Dr. Jack's methods. But where there were no proximate teeth, he had found that it consumed too much time to make vulcanized rubber appliances, as recommended by Dr. Jack, and this had led him to the making of experiments for the purpose of securing something more easily adjustable, and which should secure the same ends as the appliances which must be specially made for each case. The matrix which he has finally designed, is in the form of a band made of thin spring-tempered steel, and which is therefore easily adapted to the irregular form of any tooth upon which it may be placed. The band is doubled, or made thicker upon one side, and this is penetrated by a screw, the blunt point of which rests against either the buccal or lingual wall of the tooth, and when it is set up by a watch-key, which fits the projecting end of the screw, the band is firmly fixed in place and assumes the natural contour of the tooth, thus enabling the missing walls to be restored exactly. They are specially adapted to the molar and bicuspid teeth upon the approximal surfaces. The band is so thin that it is readily inserted between teeth that may be in close proximity, and thus the time and annoyance so frequently attending the wedging of posterior teeth is saved the patient and operator. In the upper teeth the screw should be set on the buccal surface, while in the lower teeth it should be placed against the lingual wall, as these surfaces approach more nearly to the perpendicular, and there is, therefore, less danger of slipping.

In filling approximal cavities, which dip deep down under the gum, it is often difficult to keep the rubber dam in position. For these cases he has devised an annex to the band matrix, which consists of a separate piece of the thin steel folded upon itself and slipped upon the inferior edge of the matrix. After the matrix is approximately in position this is crowded down over the depression of the cavity, carrying with it the rubber-dam. The screw is then tightened and the whole thus retained in position.

When a considerable portion of the buccal or lingual wall has been lost, the thin matrix will sometimes draw into the cavity when the screw is set up. In these cases Dr. Brophy uses another annex made of German silver bent to the shape of the tooth, set within the matrix and held firmly in position when the screw is set up. The lower edge of the annex may be turned outward, so that it will be readily crowded down into position by the matrix.

Dr. Brophy's Band Matrix and Annex.

When the rubber-dam and matrix are in position, the filling of cavities which involve one or more of the walls of a tooth is very much simplified. He commences with a large pellet of soft gold, or of tin and gold, and carries it between the matrix and the cervical wall of the tooth. It is thus held firmly in position until it is thoroughly malleted home, and the cervical edge thus made secure. Another piece is added and the cavity thus partially filled, when cohesive gold is used to give a firmer and harder masticating surface. He finishes the whole with No. 60 cohesive gold. For the insertion of the soft, or non-cohesive gold, he uses a wedge-shaped plugger that was first presented to his notice by Dr. Benjamin Lord, and this he considers the most valuable instrument for the manipulation of soft gold, or gold and tin, that has yet been devised. This plugger has deep serrations upon the point and on each side.

The band matrix will yield sufficiently to allow for a little overlapping of the walls of the cavity, and by loosening the screw this may be increased to any extent.

Dr. Brophy believes that soft, or non-cohesive gold, is preferable for the cervical walls of cavities and for the first half of all fillings, because of its more perfect adaptation. Gold and tin, in consequence of its ease of manipulation and excellent adaptability, form an excellent material with which to fill the base of large approximal cavities in posterior teeth.

The band matrix is very simple, and by its use and that of the different annexes, many cavities, which have hitherto presented extreme difficulties in their successful filling, are reduced to simple ones, and the operation is made much less formidable, while the results in his hands have been far better than under the old method.

*Dr. Dwinelle*—Stated that he had, years ago, used a kind of matrix in filling approximal cavities, but was glad to have the employment of the matrix reduced to a system, and he thanked Dr. Brophy for this design.

*Dr. Perry*—Used a very narrow matrix of phosphor-bronze at the cervical wall, to be held in position by his separators. He does not care to use them very wide, as he is not sure of getting the filling perfect at the walls. The doctor thinks where the matrix is employed extra care must be used in filling. He also exhibited to the society a very neat and effective matrix designed by his associate, Dr. Woodward.

*Dr. Clowes*—Wished to call the society's attention to two cases, which he had not the opportunity of doing earlier in the evening under the head of "Incidents of Office Practice." First, a young lady twenty-one years of age, with an extremely handsome set of teeth, the result of "oral gardening" was taken sick, and remained so for three weeks. On recovery the teeth were greatly pitted, and presented soft white spots. These were carefully cut out and filled with gold. At the expiration of six months the edges of the fillings showed softening, and these spots were cut out, where required, and gold added. In another six months the same condition reoccurred, when the gold was taken out and the teeth filled with oxy-phosphate. This, in a short time, washed out, and as a last resort the fillings were again removed and the cavities filled with amalgam, since which no white or softened spots have appeared.

Case second was a lady fifty years of age, with the cutting edges of her teeth filled with gold. These were imperfect, and being anxious to save the teeth at any cost, she was advised that they be cut out and filled with amalgam. Though probably causing criticism for placing amalgam in such a position in the front teeth, the doctor stated it as his belief that this only would save the teeth. The operation was performed, and the result is all that could be desired, and the patient well pleased.

*Dr. Andrews*—Of Cambridge, Mass., was greatly interested in the paper of the evening. He had used as a matrix thin strips of copper bound to the tooth by floss silk, after the rubber dam had been adjusted. Some of these strips he had silver plated, thus acting as a reflector of light.

*Dr. S. B. Palmer*—Of Syracuse, thought in using gold and tin combined as a filling, great care should be taken in its preparation. He thought the metals should be folded, and not used in a rope, as when introduced into a cavity if a thick layer of each be placed intermediate, the filling became in time pitted or roughened, while when folded in thin alternate sheets, there was some chemical action which took place, and in time it made a solid and lasting filling.

*Dr. Brockway*—Referring to filling with the matrix, stated that he often put amalgam at the cervical wall and then filled with gold.

THE ANNUAL DINNER OF THE SOCIETY.

The banquet hall of the Brunswick Hotel, Fifth Avenue, New York City, was the scene of the annual dinner on the evening of February 10th. The menu was an excellent one, and the display of flowers and other table ornaments very beautiful.

Among the invited guests who were not able to be present may be mentioned ex-President Arthur, ex-Govs. Hoffman and Cornell, Hon. Carl Schurz, Rev. Henry Ward Beecher, Oliver Wendell Holmes and many others.

The after-dinner speaking was opened briefly by Dr. Bogue, the president, who welcomed the guests, indulging in a few witty personal allusions, and said that the guests of the society had demonstrated the good quality of the dentists' work by the facility with which they had demolished the good things on the menu. Dr. N. W. Kingsley was then introduced as toastmaster of the evening. Dr. Kingsley was not prepared with any formal toast list, and began reading several letters of regret from those who had been invited but could not come.

Dr. Oliver Wendell Holmes wrote: "I often think of the forlorn condition of some of the great personages of history in the days when there were no dentists, or none who would be recognized as such by the dental artists of to-day. I think of poor King David, a worn-out man at seventy, probably without teeth and certainly without spectacles. Think of poor George Washington, his teeth always ready to drop like a portcullis and cut a sentence in two. See him in Stuart's admirable portrait, his thoughts evidently divided between the cares of empire and the maintenence of *status in quo* of his terrific dental arrangements. Think of Walter Savage Landor's melancholy complaint that he did not mind losing his intellectual faculties, but the loss of his teeth he felt to be a very great calamity. I will propose, then, the dental profession, and this association as its worthy representative It has established and prolonged the reign of beauty; it has added to the charms of social intercourse and lent perfection to the accents of eloquence; it has taken from old age its most unwelcome feature, and lengthened enjoyable human life far beyond the limit of the years when the toothless and poor blind patriarch might well exclaim, 'I have no pleasure in them.'"

Another letter of regret was received from Henry Ward Beecher, but Dr. K. thought he would have to be allowed some time to spell it out; Mayor Grace, Gov. Hoffman, Hon. Hamilton Fish, Judge Daly, Gen. Stewart Woodford, John Bigelow, T. Gaillard Thomas, Dr. Elisha Tucker, and Dr. McDonald of the Ward's Island Insane Asylum, also sent expressions of regret at their inability to be present.

The toast of the evening was "The Odontological Society." Dr. J. Smith Dodge, Jr., of New York, responded, saying that "the keynote of these festivities was to be found in the proceedings of the regular meeting of the evening before." Dr. Dodge made an eloquent plea for dentistry as a high calling, and closed with an admirable peroration.

Gen. Horace Porter, formerly of Gen. Grant's staff, was introduced as a "son-of-a-gun" from New York, and asked to tell what he knew of dentistry from the patient's standpoint. He made one of his characteristic after-dinner speeches, full of points and puns, and said it was the first time that he had fallen into the hands of dentists and found his mouth in condition for speech-making. He said, referring to Dr. Bogue, "He has treated me for a long time, not from motives of pure friendship, but, as they say in tariff discussions, for purposes of revenue only. Of late years I have never heard his name mentioned without feeling for my teeth to see if they were all there."

Rev. Dr. Howard Crosby, of New York, said he and most patients would pronounce the name of the Society as the "Oh-don't-ological," but he would inveigh against the snobbery which would rank above a dentist the man who did nothing useful in the world.

Prof. Garrettson, Dr. Wm. H. Atkinson, and the Rev. Dr. A. F. Beard, of Paris, followed with timely speeches.

Rev. Dr. Beard, of Paris, said that without teeth there was little enjoyment. Also, that a good dentist must in every respect be a gentleman, and in his practice gentleness and skill should be combined. Men of coarse nature are not fit for such duties, and cannot succeed. The superiority of American dentists is recognized all over the world, and those who have gone from this country have achieved reputations abroad. Their ability is acknowledged, and they are consulted and conferred with. Dentists alleviate pain and give comfort to t thousands.

Allusion was made by the chairman to the illness of Dr. Dwinelle during the season of the annual dinner last year, and after congratulating the doctor on his recovery and his ability to be present on this occasion, the speaker proposed to drink to the health of Dr. Dwinelle.

Dr. Dwinelle thanked the gentlemen present in a most feeling manner for their sympathy, etc., so kindly expressed.

Dr. L. D. Shepard expressed his gratification in listening to remarks of previous speakers. He considers dentists, if true to their calling, peers in any walk of life. They give artistic beauty to deformed faces, and administer much to the health and physical well-being of man.

Dr. Kingsbury had long looked upon the Odontological Society as a teacher. He also was a teacher, and one of the founders of Philadelphia College. Dr. K. referred to the unusual mortality of prominent men of our profession, paying a tribute to the memories of Drs. Barnum and Riggs. He also spoke of the late Dr. Horace Wells as the discoverer of anasthesia, and that through him Dr. K. had become a dentist. He felt it pleasant to be here and away from his office, stating also that dentists are too much confined, get into sedentary habits and have too much nervous excitement.

They should take exercise and enjoy life. He recommended angling in particular, and recited two poems descriptive of such sport.

Dr. Crouse visited New York, partly to see the New York dentists in their offices. He desired to know something of their methods, their instruments and appliances. He had called on a number, and he intended to call on many others. He finds that New York dentists make a display of wealth, but Chicago dentists pay their debts and make less show. He finds New York a very muddy city, yet he suggested the idea of annexing it to Chicago. In Chicago they have snow instead of mud, and for pastime or recreation they throw snow balls, so do not find it necessary to go fishing for sport.

The company rose from the table at 12.30 A. M., and the festivities came to an end.

## Figures and Tables

**Figure f1:**